# Hyaluronic acid-based bioink improves the differentiation and network formation of neural progenitor cells

**DOI:** 10.3389/fbioe.2023.1110547

**Published:** 2023-03-03

**Authors:** Inês Pereira, Maria J. Lopez-Martinez, Aranzazu Villasante, Clelia Introna, Daniel Tornero, Josep M. Canals, Josep Samitier

**Affiliations:** ^1^ Nanobioengineering Group, Institute for Bioengineering of Catalonia (IBEC), The Barcelona Institute of Science and Technology (BIST), Barcelona, Spain; ^2^ Department of Electronic and Biomedical Engineering, University of Barcelona, Barcelona, Spain; ^3^ Biomedical Research Networking, Center in Bioengineering, Biomaterials and Nanomedicine (CIBER-BBN), Madrid, Spain; ^4^ Laboratory of Stem Cells and Regenerative Medicine, Department of Biomedical Sciences, Faculty of Medicine and Health Sciences, Institute of Neurosciences, University of Barcelona, Barcelona, Spain; ^5^ Creatio - Production and Validation Center of Advanced Therapies, Faculty of Medicine and Health Sciences, University of Barcelona, Barcelona, Spain; ^6^ Research Foundation Clinic Barcelona-August Pi i Sunyer Biomedical Research Institute (FRCB-IDIBAPS), Barcelona, Spain; ^7^ Laboratory of Neuronal Stem Cells and Cerebral Damage, Department of Biomedical Sciences, Faculty of Medicine and Health Sciences, Institute of Neurosciences, University of Barcelona, Barcelona, Spain

**Keywords:** bioprinting, biomaterials, *in vitro* models, neural progenitor cells, differentiation, neuronal models

## Abstract

**Introduction:** Three-dimensional (3D) bioprinting is a promising technique for the development of neuronal *in vitro* models because it controls the deposition of materials and cells. Finding a biomaterial that supports neural differentiation *in vitro* while ensuring compatibility with the technique of 3D bioprinting of a self-standing construct is a challenge.

**Methods:** In this study, gelatin methacryloyl (GelMA), methacrylated alginate (AlgMA), and hyaluronic acid (HA) were examined by exploiting their biocompatibility and tunable mechanical properties to resemble the extracellular matrix (ECM) and to create a suitable material for printing neural progenitor cells (NPCs), supporting their long-term differentiation. NPCs were printed and differentiated for up to 15 days, and cell viability and neuronal differentiation markers were assessed throughout the culture.

**Results and Discussion:** This composite biomaterial presented the desired physical properties to mimic the ECM of the brain with high water intake, low stiffness, and slow degradation while allowing the printing of defined structures. The viability rates were maintained at approximately 80% at all time points. However, the levels of *β*-III tubulin marker increased over time, demonstrating the compatibility of this biomaterial with neuronal cell culture and differentiation. Furthermore, these cells showed increased maturation with corresponding functional properties, which was also demonstrated by the formation of a neuronal network that was observed by recording spontaneous activity *via* Ca^2+^ imaging.

## Introduction

Due to the difficulty in obtaining samples of the human brain at different stages of development, many associated mechanisms remain unelucidated (Kajtez et al., 2021). This hinders the treatment of brain diseases, which are primarily difficult to be cured. The use of conventional 2D *in vitro* and animal models is the standard practice to study the brain, and although breakthroughs were possible, they still have drawbacks. The lack of complexity, difficulties in multiple co-cultures in 2D cultures, and increased economic and ethical costs of animal models are the most important disadvantages, thereby reinforcing the need for improved models to study the brain and its diseases (Choi, Lee, and Jeong, 2022). In the last few years, three-dimensional (3D) models have emerged as the future of *in vitro* modelling. They incorporate biomaterials to provide a scaffold for cells to interact with each other and the extracellular matrix (ECM). Compared to *in vivo* conditions, this change in the architecture has resulted in similar cell morphology, proliferation rates, and differentiation conditions (Baharvand et al., 2004; Liu et al., 2006; Soares et al., 2012; Duval et al., 2017).

Three-dimensional bioprinting has been explored in *in vitro* modelling for its ability to control the precise deposition of materials and cells, allowing the production of models that can possess the architecture and cell distribution of the natural tissue (Knowlton et al., 2018). Gelatin and alginate hydrogel-based bioinks are used in bioprinting because they have elastic and variable mechanical properties, such as pore size and stiffness, which can be adapted to the encapsulation of neuronal cells (Xiao et al., 2019; Poorna et al., 2021). Gelatin is a naturally sourced polymer that is biocompatible and biodegradable. Furthermore, it possesses natural arginine–glycine–aspartic acid (RGD) sequences that aid in cell attachment. In addition, it has sequences for matrix metalloproteinase (MMP) binding and cleavage, which allow cell mobility and remodelling, and is usually chemically modified to incorporate methacrylate groups to better control the cross-linking of the material (Yue et al., 2015). In contrast, alginate is widely used as a bioink in neural tissue engineering models (Ning et al., 2019; Xiao et al., 2019; Sachdev et al., 2022). It is a polysaccharide found in seaweed. It is biocompatible and non-immunogenic, and is not degraded by cells. The mechanical properties of alginate, such as stiffness and porosity, can be tuned by changing its concentration or conjugation with different ligands or materials. This can be beneficial for adapting hydrogels according to the properties of the ECM (Neves et al., 2020). The combination of gelatin and alginate has been explored for bioprinting applications, particularly those involving the modelling of different tissues of the body, because the balance between degradable gelatin and non-degradable alginate can aid in developing long-lasting models (Chen et al., 2020). Hyaluronic acid (HA) is another commonly used material because it is one of the most abundant molecules in the brain and is capable of regulating cell adhesion, migration, and differentiation (Khaing and Seidlits, 2015). HA is difficult to be used in printing because of its poor mechanical strength, but it can be functionalized or mixed with other materials such as gelatin and alginate (Ouyang et al., 2016; Noh et al., 2019; Kajtez et al., 2021; Ding et al., 2023).

In this study, a composite material composed of gelatin methacryloyl (GelMA), methacrylated alginate (AlgMA), and HA was explored as a suitable bioink configuration for the extrusion bioprinting of NPCs and to support differentiation processes. The mechanical properties were evaluated in terms of cell viability, differentiation, and compatibility of the composite hydrogel for use as an extrusion bioprinting bioink. GelMA + AlgMA + HA presented a high capacity for water intake and low stiffness, which was compatible with the culture and differentiation of NPCs for up to 28 days.

## Materials and methods

### Synthesis of the polymers

Gelatin (G1890, Sigma-Aldrich, USA) was altered to obtain a 40% methacrylation degree as described previously (Visser et al., 2015). In brief, gelatin was dissolved at a concentration of 10% (w/v) in 10 mM PBS (P4417, phosphate-buffered saline, Sigma-Aldrich, USA), and methacrylic anhydride (276685, Sigma-Aldrich, USA) was added dropwise with constant stirring. After 1 hour, 10 mM PBS was added to stop the solution from diluting to 5x. The solution was then dialyzed against Milli-Q water in 3.5 kDa SnakeSkin membranes (Thermo Fisher Scientific, USA) for 3 days at 40°C. The final solution of gelatin methacryloyl was lyophilized and stored at −20°C. Methacrylation of sodium alginate was performed as described earlier (Kloxin et al., 2010). In brief, sodium alginate (1% w/v) (W201502, Sigma-Aldrich, USA) was dissolved in 50 mM MES buffer at pH 6.5 as well as 20 mM EDC (N-(3-dimethylaminopropyl)-N-ethylcarbodiimide hydrochloride) (39391, Sigma-Aldrich, USA) and 10 mM N-hydroxysuccinimide (130672, Sigma-Aldrich, USA). Subsequently, 10 mM 2-aminoethyl methacrylate hydrochloride was added for 10 minutes (516155, Sigma-Aldrich, USA). After incubation for 24 h at 40 °C, acetone (131007, Panreac, Spain) was used to stop the reaction, and the solution was filtered through a vacuum flask. Milli-Q water was used to dissolve the precipitate, which was filtered. The final solution of AlgMA was dialyzed, lyophilized, and stored, as described for gelatin methacrylation.

### Preparation of the hydrogels

Different formulations of hydrogels were produced with 3%–5% (w/v) GelMA, 0%–1% (w/v) AlgMA, and 0%–5% HA (600-01-02, 15kD–30kD; Contripo, Czech Republic) (Table 1). Low-molecular weight HA was selected owing to its promotion of cell proliferation, differentiation, and migration while allowing easier solubilization of the formulation (Moshayedi and Carmichael, 2013). The final formulation characterized in this study comprised 5% GelMA +1% AlgMA +1.5% HA, with a control formulation of 5% GelMA +1% AlgMA. The polymers were diluted in PBS 10 mM for physical characterization or in cell proliferation medium for biological assays at 40 °C overnight. Then, 0.05% of the photoinitiator lithium phenyl-2,4,6-trimethylbenzoylphosphinate (LAP) (L0290, TCI Europe N.V, Belgium) was added to the hydrogel. The hydrogels were then cross-linked for 5 s using a 3D bioprinter (3DDiscovery BioSafety, REGENHU, Switzerland; 365 nm, 3 W cm^−2^) with a UV light source.

**TABLE 1:** Different formulations of hydrogels used in the optimization process.

GelMA (%)	AlgMA (%)	HA (%)
3	0	2.5
3	0	5
3	0.5	2.5
3	0.5	5
5	1	0
5	1	1.5

### Physical characterization of the hydrogels

#### Swelling analysis

The polymers were mixed as previously described, and 300 µL of the solution was placed in a 48-well plate and exposed to UV light. The samples were then removed from the plate, weighed, and placed in a 24-well plate with 10 mM PBS. Weight was then measured at 15-min intervals for the first hour, and then at 2, 4, 6, 8, 24, and 48 h. The wet weight increase ratio (∆W) was calculated using the following equation:

$$∆W=\frac{Ws-Wi}{Wi}∙100$$ (1)

Here, Ws represents the weight after swelling and Wi is the initial weight of the sample. The mass increase was normalized to the initial weight of the sample.

#### Degradation analysis

For this analysis, the fabricated samples were placed in a 12-well plate with 10 mM PBS for 24 h after removal from the 48-well plate. Collagenase type II (17101015. Thermo Fisher, USA) was added (1.5 U mL^-1^) and the samples were incubated at 37 °C under conditions involving shaking. The samples were weighed for 15 min in the first hour, and then at 2, 3, and 4 h. The percentage of the remaining sample (%Wr) was calculated using the following equation:

$$\%Wr=\frac{Wt}{Wi}∙100$$ (2)

Here, Wt represents the weight of the samples after incubation and Wi is the initial weight.

#### Evaluation of compression modulus

For the uniaxial compression test of the hydrogels, samples were prepared as previously described in a 48-well plate and transferred to a 12-well plate with 10 mM PBS. After 24 h in PBS, the samples were cut using a 10-mm-diameter biopsy punch, and the real diameter and height were measured. The samples were then tested with a Zwick Z0.5 TN instrument (Zwick-Roell, Germany) using a 5N load cell at room temperature until 30% deformation was achieved (0.1 mN of preload force and strain rate of 20% min^−1^). The compressive modulus was determined by extracting the slope of the linear region in the interval of 10%–20% deformation.

#### Scanning electron microscopy

The hydrogel samples were prepared as described in the compression tests. After 24 h in PBS, the samples were fixed with glutaraldehyde (2.5% diluted in 0.1 M PBS, pH 7.4, G6257, Sigma-Aldrich, USA) for 2 h. The samples were then dehydrated by immersion in graded ethanol solutions in Milli-Q water: 50% (once for 10 min), 70% (twice for 10 min), 90% (thrice for 10 min), 96% (thrice for 10 min), and 100% (thrice for 10 min). The samples were then placed in a critical point dryer (Leica EM CPD300, Austria) and imaged using ultrahigh-resolution scanning electron microscopy (Nova NanoSEM 230, FEI Company, Netherlands). On hydrogels with cells, the samples were fixed and dehydrated at the respective time points using the same protocol, followed by critical point drying and imaging.

### Cell culture

Mouse NPCs C17.2 (Snyder et al., 1992) (provided by Dr. Evan Y. Snyder, Department of Neurology and Pediatrics, Harvard Medical School and Division of Neuroscience, Children’s Hospital, Boston, MA) were expanded in the proliferation medium [Dulbecco Modified Eagle Medium (DMEM) high glucose, L-glutamine (41965039, Gibco, Thermo Fisher, USA) supplemented with 10% fetal bovine serum (10270106, Thermo Fisher, USA), 5% horse serum (26050088, Gibco, Thermo Fisher), 1% antifungal-antimitotic solution (15240062, Gibco, Thermo Fisher, USA), and 1% GlutaMAX (35050-061, Gibco, Thermo Fisher, USA)]. The differentiation medium was composed of DMEM:F12 (11320074, Gibco, Thermo Fisher, USA) supplemented with 1% N-2 supplement (17502048, Life Technologies), 1% antifungal-antimitotic solution (15240062, Gibco, Thermo Fisher, USA), 10 ng mL-1 NGF (13290010, Life Technologies, Thermo Fisher, USA), and 10 ng mL-1 BDNF (450-02, Peprotech Inc, USA), and added 1 day after inclusion in the biomaterial.

### Inclusion of C17.2 cells in the composite hydrogel

**FIGURE 1:**
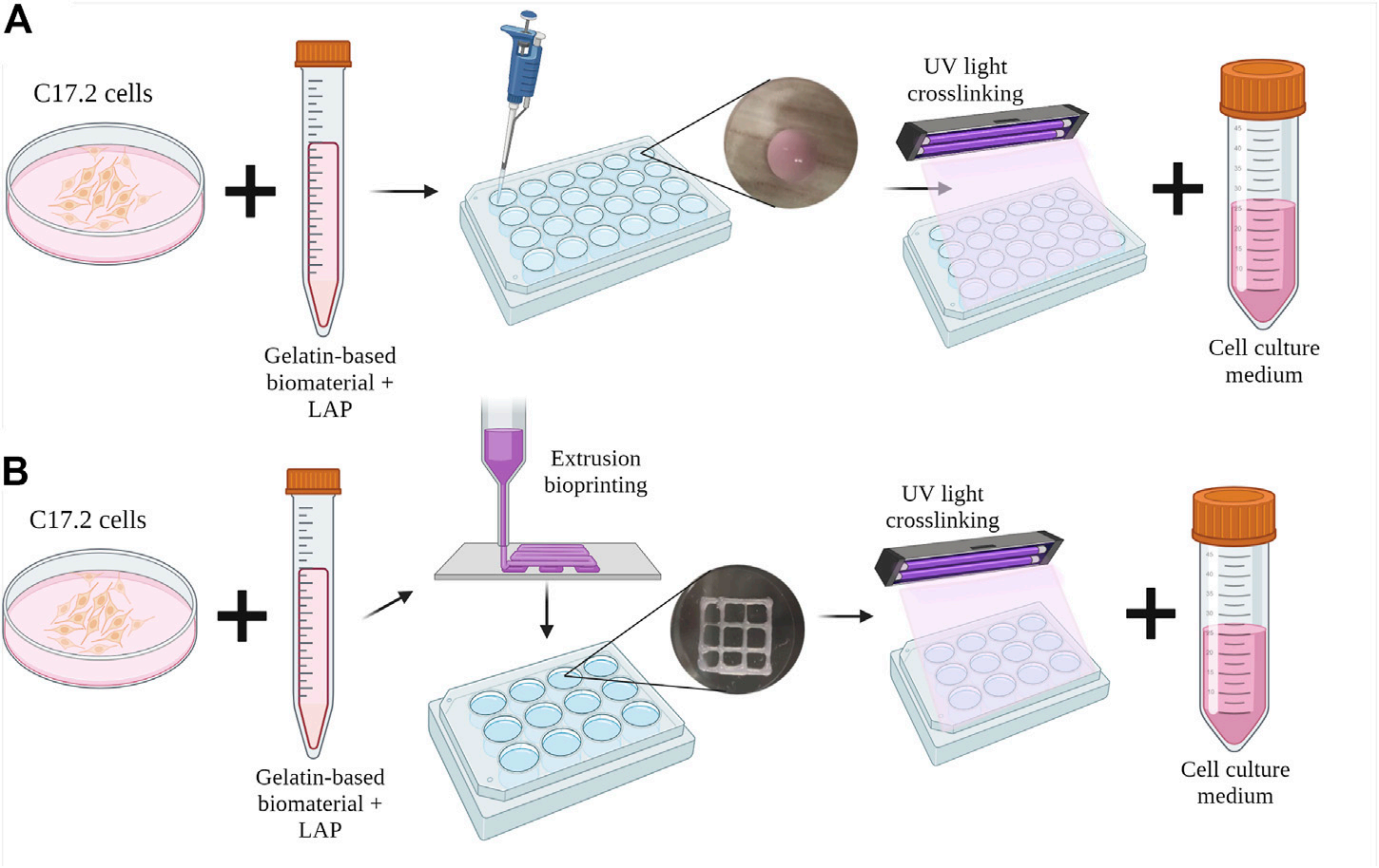
Schematic diagram of the inclusion of C17.2 cells in the hydrogel **(A)** in the drop-shaped samples and **(B)** in extrusion bioprinted samples. Created with Biorender.com.

C17.2 cells were mixed at a density of 1 × 10^6^ cells/mL with the dissolved polymer precursors and photoinitiator LAP after overnight dissolution. After mixing, 30 µL drops were plated in a 24-well plate, and each well was irradiated for 5 s using a 3D bioprinter (3DDiscovery BioSafety, REGENHU, Switzerland; 365 nm, 3 W cm−2) with a UV light source (Figure 1A). The cell proliferation medium was added to each well and replaced with the differentiation medium on the next day (day 1). The medium was replaced every 2–3 days until day 28 of culture.

### Bioink preparation and extrusion bioprinting parameters

After overnight dissolution in the cell proliferation medium, the polymers were mixed with C17.2 cells at a density of 1 × 10^6^ cells mL^-1^ and the photoinitiator LAP. The bioinks were then placed in a 3-cc printing syringe (Nordson Corporation, USA) and loaded into the direct dispensing head of the bioprinter (3DDiscovery BioSafety, REGENHU Ltd, Switzerland; 365 nm, 3 W cm−2) with a cooling chamber at 18 °C. Squares (10 mm) with an internal grid were designed with BioCAD v1.0 software (REGENHU Ltd, Switzerland) and converted to computer-aided design (CAD) files, which were opened in the 3D Discovery HMI software interface (REGENHU Ltd, Switzerland). The bioinks were printed in two layers *via* a 200-µm nozzle at a rate of 30 mm s^-1^ with 2 Pa pressure. During optimization, the printing parameters varied with temperature and printing speed (Supplementary Table S1). After each of the two layers was printed, the sample was irradiated with UV light (365 nm) for 5 s, and then the cell proliferation medium was added to the wells (Figure 1B). The following day, the medium was replaced with the differentiation medium every 2–3 days until day 15 of culture.

### C17.2 viability assessment through a live/dead assay kit

Cell viability was assessed using a live/dead assay kit (L3224, Thermo Fisher, USA) comprising calcein AM and ethidium homodimer (EthD-1). After washing with 10 mM PBS, the samples were incubated with 1% v/v calcein, 1% v/v EthD-1, and 1% v/v Hoechst (H3570, Life Technologies, Thermo Fisher, USA) for 20 min at 37 °C. Then, another three washes with PBS were performed, and the samples were examined under a confocal microscope (SP5 Leica, Austria) where fluorescence images were captured at 20 x. The percentage of cell survival was calculated by counting the number of live cells using MATLAB^®^ software.

### Evaluation of protein expression *via* the fluorescence immunofluorescence assay

The samples were fixed and stained to evaluate the expression of proteins involved in the differentiation process of C17.2 cells at different time points until day 28 of differentiation. In brief, the samples were washed 2–3 times with PBS, fixed with 4% paraformaldehyde (P6148, Sigma-Aldrich, USA) for 15 min, and then washed 2–3 times additionally with PBS for 10 min. The samples were then permeabilized with 0.1% v/v Triton X-100 (T8787, Sigma-Aldrich, USA) and 5% v/v FBS for 2 h. Three washes with PBS containing 0.1% Triton^®^ X-100 were performed, and the samples were incubated overnight with the primary antibody solution containing anti-nestin antibody (1:250; MBS500041; MyBioSource, USA) and TUJ1 antibody (1:1000; ab18207; Abcam, UK) at 4 °C with shaking. The secondary antibody solution containing goat anti-mouse Alexa Fluor 488 (1:1000; 10667; Invitrogen, Thermo Fisher, USA) and goat anti-rabbit Alexa Fluor 568 (1:1000; 11011; Invitrogen, Thermo Fisher, USA) was added after three washes with PBS-Triton 0.1%, followed by overnight incubation at 4 °C under conditions involving shaking and protection from light. Next, the samples were washed thrice with PBS, and 1% Hoechst was added for 15 min, followed by three washes with PBS. The samples were then observed under a confocal microscope.

### Gene expression through qRT-PCR

TRIzol™ reagent (15596018, Thermo Fisher, USA) was used to mechanically homogenize the samples at three different time points of culture (days 1, 15, and 28). RNA was extracted according to the manufacturer’s protocol. In brief, chloroform (C2432, Sigma-Aldrich, USA) was used for phase separation, followed by 2-propanol to precipitate RNA. The pellet was washed with ethanol and dissolved in nuclease-free water (R0581, Sigma-Aldrich, USA). cDNA retrotranscription was performed using Ready-To-Go You-Prime First-Strand Beads (27926401; GE Healthcare, USA). Polymerase chain reaction (PCR) solutions were prepared using PowerUpTM SYBRTM Green Master Mix (A25742, Applied Biosystems, USA). The gene expression was evaluated using the StepOnePlusTM Real-Time System (Applied Biosystems, USA), and the expression was calculated with the following formula: (ΔC। GADPH -ΔC। Sample) x10^4^. Mouse primer sequences used are described in Table 2 and were extracted from the PrimerBank Database (https://pga.mgh.harvard.edu/primerbank/).

**TABLE 2:** Genes used in the qRT-PCR experiments.

Gene	PrimerBank ID
Nestin	15011851a1
β-III Tubulin	12963615a1
MAP2	68341934c3
GFAP	196115326c1
PAX6	346644711c1
DCX	46575787c1
S100b	6677839a1

### Functional evaluation through calcium imaging recordings

Calcium imaging recordings were performed to assess neuronal activity in the cultures. On the day of the recording, the cell medium was removed, and samples were washed with artificial cerebrospinal fluid solution (aCSF) (0.1 M Hepes (H4034, Life Technologies, Thermo Fisher, USA), 128 mM NaCl (131659, Panreac, Spain), 4 mM KCl (60142, Sigma-Aldrich, USA), 10 mM glucose (G8769, Sigma-Aldrich, USA), 45 mM sucrose (107651, Merck, USA), 2 mM CaCl_2_ (C3306, Sigma-Aldrich, USA), and 1 mM MgCl_2_ (M8266, Sigma-Aldrich, USA) dissolved in Milli Q water (pH 7.4). Then, the samples were placed in glass bottom chambers with a diameter of 35 mm (MatTek Corporation, USA) and incubated with 2 mL of aCSF containing 2 µM Fluo4-AM (Life Technologies, Thermo Fisher, USA) for 15 min at 37 °C. The samples were washed again with aCSF to remove the excess fluorescent dye and placed under a fluorescence microscope (Nikon Eclipse Ti2, Nikon Instruments Inc, USA). The images were recorded for 10 min under a ×20 objective with a frame rate of 2.2fps. Calcium recordings were analyzed using the custom-made software NETCAL (Orlandi et al., 2017), by which the activity traces were extracted.

### Statistical analysis

All graphs are presented as mean and standard deviation values. Statistical analysis was performed with GraphPad using an unpaired *t*-test for the analysis of pore diameter and a two-way variance analysis (ANOVA) with Turkey’s and Sídák’s *post hoc* multiple comparisons tests for all other analyses. However, only Sídák’s *post hoc* multiple comparisons test results were used to simplify the visualization. The *p*-value was set at 0.05.

## Results

### GelMA + AlgMA + HA composite hydrogel demonstrated promising physical characteristics

In this study, GelMA + AlgMA + HA and GelMA + AlgMA (as a control) supplemented with 0.05% LAP were evaluated. The polymer concentrations were previously optimized using viability and immunofluorescence assays to evaluate sample degradation (Supplementary Figure S1). Gelatin was first added at 3%, with a variation of 0%–0.515% for AlgMA and 2.5%–5% for HA. Even though the viability of the cells (Supplementary Figure S1A) was high and the cells started to differentiate (Supplementary Figure S1B), the samples degraded over the course of 8 days (Supplementary Figures S1C, D). To avoid this degradation, the amount of GelMA was increased to 5% and that of AlgMA was increased to 1%. In the samples with a higher percentage of HA in the optimization process, the sample degraded faster than the remaining solutions. The HA concentration was reduced to 1.5%. Gelatin and alginate were methacrylated to achieve fast photo-cross-linking, which was performed by exposure to UV light for 5 s. The cross-linking time was selected based on previous studies, in which higher exposure times resulted in a significant decrease in cell viability (García-Lizarribar et al., 2018).

**FIGURE 2:**
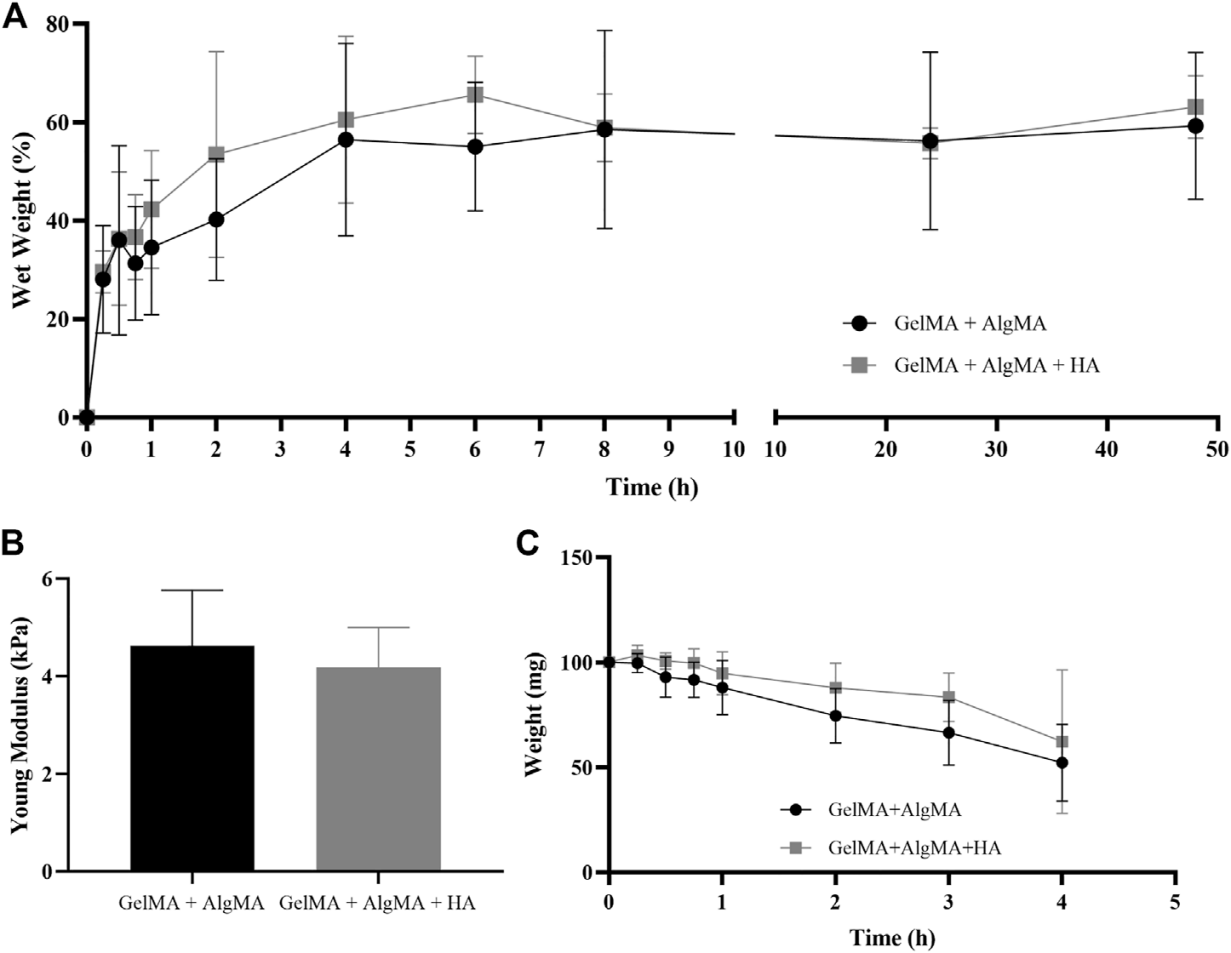
Physical characterization of the study formulation GelMA + AlgMA + HA and comparison with the control formulation of GelMA + AlgMA by examination of the results of **(A)** swelling, **(B)** compression, and **(C)** degradation assays.

The physical characterization was performed using swelling, compression modulus, and degradation assays (Figure 2). In the swelling assay, samples increased in weight up to the time point of 8 h, where they almost reached their maximum weight, and the curve was flattened (Figure 2A). In the first formulation of GelMA + AlgMA, the maximum volume increase was 59.2% ± 14.9%. For the compression modulus values, GelMA + AlgMA presented a value of 4.6 ± 1.1 kPa, whereas GelMA + AlgMA + HA had a slightly lower value of 4.2 ± 0.8 kPa (Figure 2B). To study the degradation of the hydrogels, collagenase II was added, showing that the formulation containing HA degraded more slowly (Figure 2C).

**FIGURE 3:**
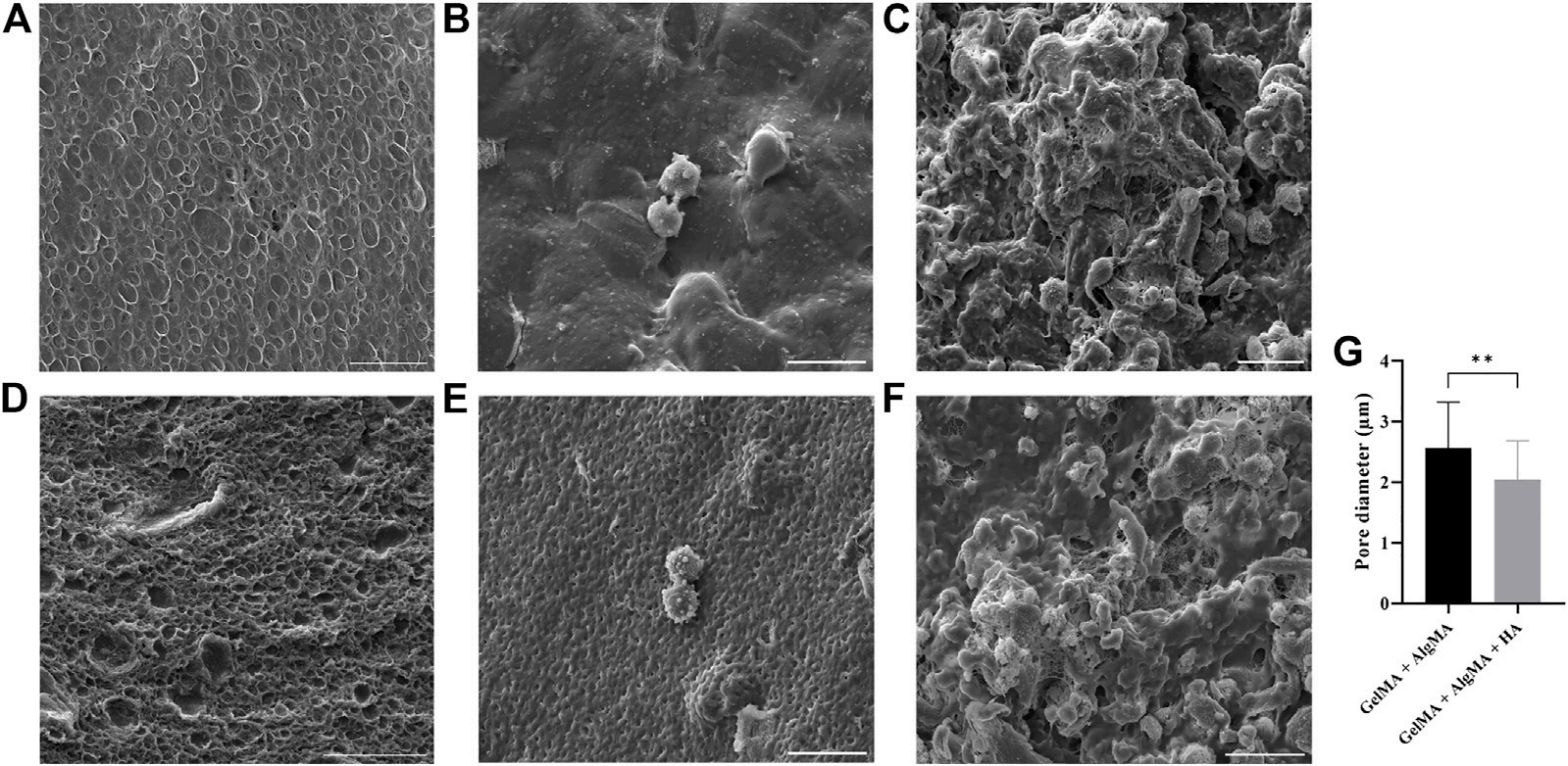
canning electron microscopy (SEM) of the hydrogels. GelMA + AlgMA without cells **(A)** on day 1 **(B)** and day 15 of culture **(C)**; GelMA + AlgMA + HA without cells **(D)** on day 1 **(E)** and on day 15 **(F)** (scale bar 20 µm); quantification of the pore diameter of both formulations **(G)**. Data are presented as mean ± standard deviation. Unpaired *t*-test statistical analysis performed: ** = *p* < 0.05.

Scanning electron microscopy (SEM) was performed on samples to evaluate the surface and porosity of our formulation and the control, as well as the cell morphology and attachment (Figure 3). GelMA + AlgMA hydrogel without cells is presented in Figure 3A, and GelMA + AlgMA + HA hydrogel is presented in Figure 3C. When these two hydrogels were compared, the formulation GelMA + AlgMA had a statistically significant higher pore diameter (2.56 ± 0.76 µm) than GelMA + AlgMA + HA (2.04 ± 0.64 µm); the quantification is represented in Figure 3G. On day 1 of the culture, GelMA + AlgMA showed dispersed cells both on the surface and underneath (Figure 3B). Similar results were observed with the GelMA + AlgMA + HA hydrogel embedded with C17.2 cells on day 1 (Figure 3E). On day 15, the cells embedded in the GelMA + AlgMA hydrogel proliferated, and aggregates were formed (Figure 3C). An arrangement of the scaffold structure was also observed because of the ECM produced by the cells. For the GelMA + AlgMA + HA hydrogel embedded with C17.2 cells on day 15, the results were identical to those of the control formulation, with increased cells, visible aggregates, and rearrangement of the scaffold (Figure 3F).

### C17.2 cells were able to survive, proliferate, and differentiate into mature neurons when embedded in the composite hydrogel

**FIGURE 4:**
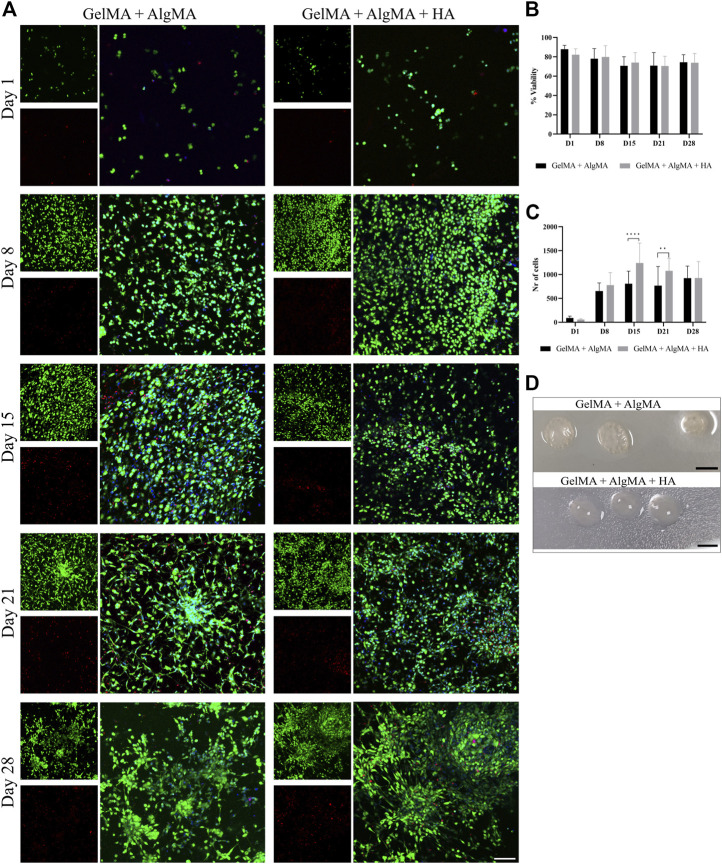
Long-term viability assay of C17.2 cells embedded in different concentrations of hydrogels for up to 28 days. **(A)** Representative confocal images of biocompatibility studies. Nuclei are stained blue, calcein AM in green represents the live cells, and EthD-1 in red represents the dead cells (scale bar 100 µm); **(B)** quantification of the viability of the cells; **(C)** quantification of the number of cells throughout 28 days in culture; **(D)** photos of the samples on day 28 (scale bar 5 mm). Data are presented as mean ± standard deviation. Statistical significance was calculated using a two-way variance analysis (ANOVA) with Sídák’s *post hoc* multiple comparisons test considering **p < 0.01; ****p < 0.0001.

For the first biological assay, the culture was maintained for up to 28 days to evaluate if the formulation would be adequate for long-term culture (Figure 4). The results showed a predominance of live cells with a rounder shape at the first two time points. After day 15, morphological changes were also more pronounced, with larger projections and increased cell connectivity (Figure 4A). These changes were most noticeable from day 15 to day 28 in our formulation, as well as in the control. The quantification of viability of the two formulations showed that most time points exhibited similar viability; all viability values were higher than 65%. However, the GelMA + AlgMA formulation showed a significant decrease in viability throughout the culture, whereas the GelMA + AlgMA + HA formulation maintained similar values of viability. The results showed that the cells were able to proliferate in all formulations, especially from day 1 to day 15, and the formulations with HA had the highest number of cells compared with the formulation without HA (Figure 4C). In terms of durability, both formulations were able to last until day 28, indicating their compatibility with long-term cultures (Figure 4D).

**FIGURE 5:**
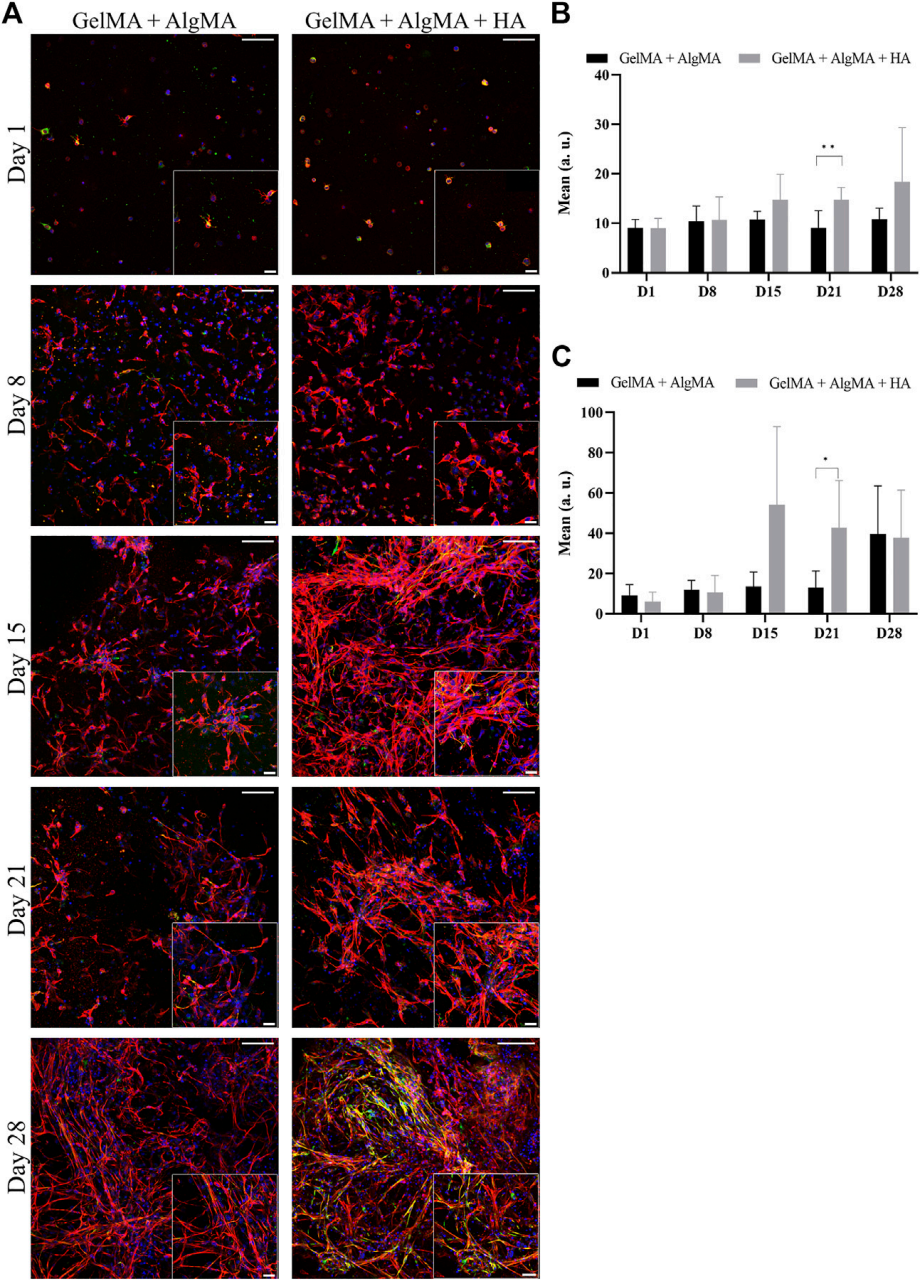
Evaluation of the differentiation of C17.2 cells embedded in different concentrations of hydrogels for 28 days. **(A)** Representative confocal images of immunofluorescence assay. Progenitor marker nestin is stained in green, neuron marker *β*-III tubulin is stained in red, and nuclei are stained in blue (scale bar 100 µm). Local zoom images are placed in the bottom right corner of each image with a scale bar of 30 µm. Quantification of the mean expression of nestin **(B)** and *β*-III tubulin **(C)** up to 28 days for both hydrogel formulations. Data are presented as mean ± standard deviation. Statistical significance was calculated using a two-way variance analysis (ANOVA) with Sídák’s *post hoc* multiple comparisons test considering *p < 0.05; **p < 0.01.

An immunofluorescent assay examining the two markers of differentiation, NPC marker nestin and early neuronal marker *β*-III tubulin, was then performed to evaluate whether our formulation was compatible with proper cell differentiation (Figure 5A). From images, changes in morphology could be observed again. This was consistent with the viability results, where an increase in cell projections and connectivity was observed from days 15 to 28. The quantification of the mean signal of the images showed that the amount of nestin was mostly consistent throughout 28 days, with a significant difference between our formulation and the control observed only on day 21; the HA formulation presented a higher mean nestin signal (Figure 5B). The expression of *β*-III tubulin increased, particularly after day 15, which was consistent with the increased projections presented by the cells (Figure 5C). The HA formulation presented a higher mean signal of *β*-III tubulin than the other formulations on day 15 along with a significant increase on day 21.

**FIGURE 6:**
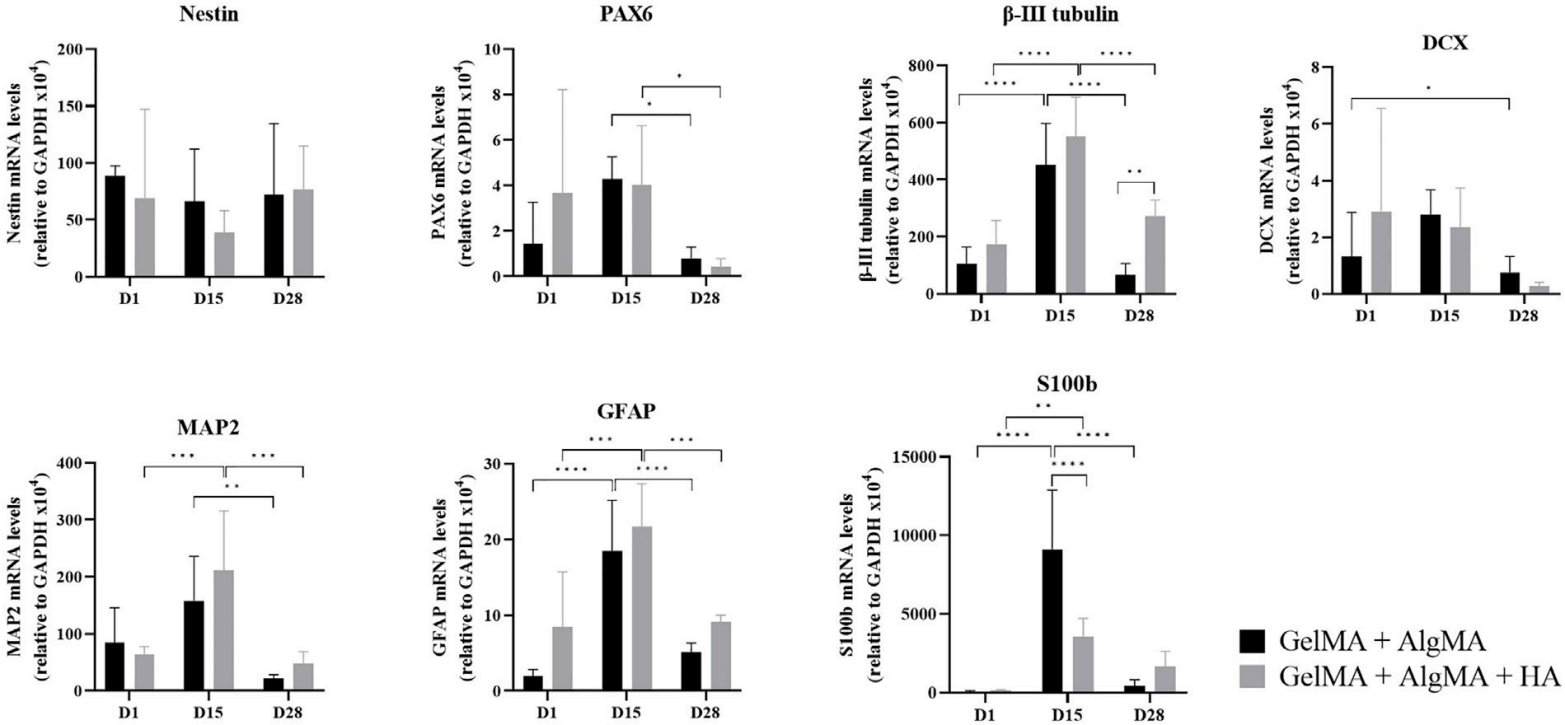
Quantitative expression of neural nestin, PAX6, β-III tubulin, MAP2, GFAP, S100b*,* and DCX genes relative to GAPDH in C17.2 cells in the two formulations on days 1, 15, and 28 of culture. Data are presented as mean ± standard deviation. Statistical significance was calculated using a two-way variance analysis (ANOVA) with Turkey’s and Sídák’s *post hoc* multiple comparisons test considering *p < 0.05; **p < 0.01; ***p < 0.001; ****p < 0.0001.

To further study the differentiation process occurring in the 3D culture, qRT-PCR was performed on GelMA + AlgMA and GelMA + AlgMA + HA samples at three different time points: days 1, 15, and 28 (Figure 6). The gene expression was normalized to that of glyceraldehyde 3-phosphate dehydrogenase (GAPDH). Two progenitor cell markers, nestin and PAX6, were also evaluated. Nestin was expressed evenly throughout the 28 days of culture. The PAX6 expression increased from day 1 to day 15 in both formulations but decreased significantly by day 28. For early neuronal differentiation, the expression of *β*-III tubulin and DCX genes was assessed. The *β*-III tubulin expression increased significantly from day 1 to day 15 on both biomaterials, whereas on day 28, it decreased significantly, even though GelMA + AlgMA + HA maintained a significantly higher expression at that time point. DCX levels remained similar on days 1 and 15 and decreased on day 28. To evaluate the maturity of differentiation, the expression of the mature neuron gene MAP2 was evaluated. The MAP2 expression, similar to *β*-III tubulin levels, increased until day 15, especially in the HA formulation but decreased significantly by day 28 in both hydrogels. As for astrocyte genes, GFAP was expressed mostly on day 15 in both formulations, while S100b was expressed significantly more in GelMA + AlgMA on day 15.

### GelMA + AlgMA + HA demonstrated suitability as a bioink with high survival of C17.2 cells

The versatility of the GelMA + AlgMA + HA hydrogel as a bioink for extrusion bioprinting was assessed. GelMA and AlgMA have been widely used as bioinks. However, the incorporation of HA could modify the properties of the hydrogel because HA is shear-thinning, which would render the bioink less viscous, and the structures could lose their definition. An optimization process was performed in which the temperature and printing speed were varied (Supplementary Figure S2; Supplementary Table S1). The first condition was not suitable for achieving a defined structure with two layers in either formulation. In the second condition, it was possible to print a two-layer structure, but with a low definition with the HA formulation and a significantly higher width of the filament (Supplementary Figures S2A, B). The third condition was suitable for printing both formulations with two layers and similar filament widths (Supplementary Figures S2, S7A).

**FIGURE 7:**
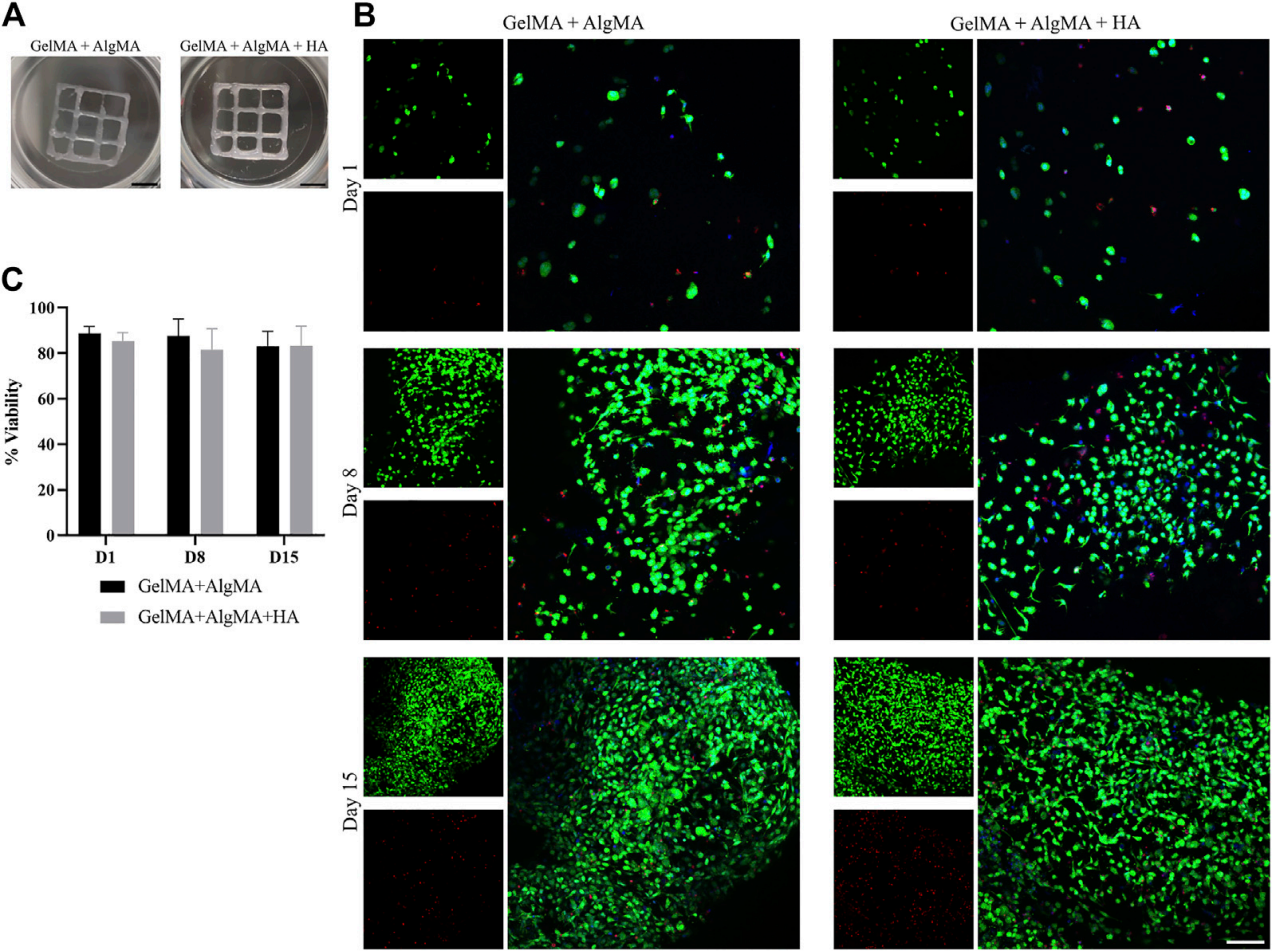
Bioprinting studies of C17.2 cells embedded in the two different bioprinted hydrogels for 15 days. **(A)** Photos of the bioprinted samples of both formulations (scale bar 2.5 mm); **(B)** representative confocal images of the viability assay. Nuclei are stained in blue, calcein AM in green represents the live cells, and EthD-1 in red represents the dead cells (scale bar 100 µm); **(C)** quantification of the viability of the cells. Data are presented as mean ± standard deviation.

The viability of the cells after printing was evaluated at three time points (days 1, 8, and 15) as before (Figure 7B). A low number of cells in the culture was observed on day 1, primarily with a round morphology and wide distances. The viability rates were 90.5% ± 4.2% for the GelMA + AlgMA formulation and 86.3% ± 4.1% for the GelMA + AlgMA + HA formulation. On day 8, increased cell number and mobility were visible, with small projections and some cell–cell contacts. The viability values of the GelMA + AlgMA and GelMA + AlgMA + HA formulations were 87% ± 7.4% and 81% ± 9.1%, respectively (Figure 7C). On day 15, proliferation increased, as well as the projections of the cells and cell connectivity. The viability values were maintained as the other time points, 82.7% ± 8.0% for GelMA + AlgMA hydrogel and 84% ± 6.9% for GelMA + AlgMA + HA.

### C17.2 cells can differentiate into neurons after printing

**FIGURE 8:**
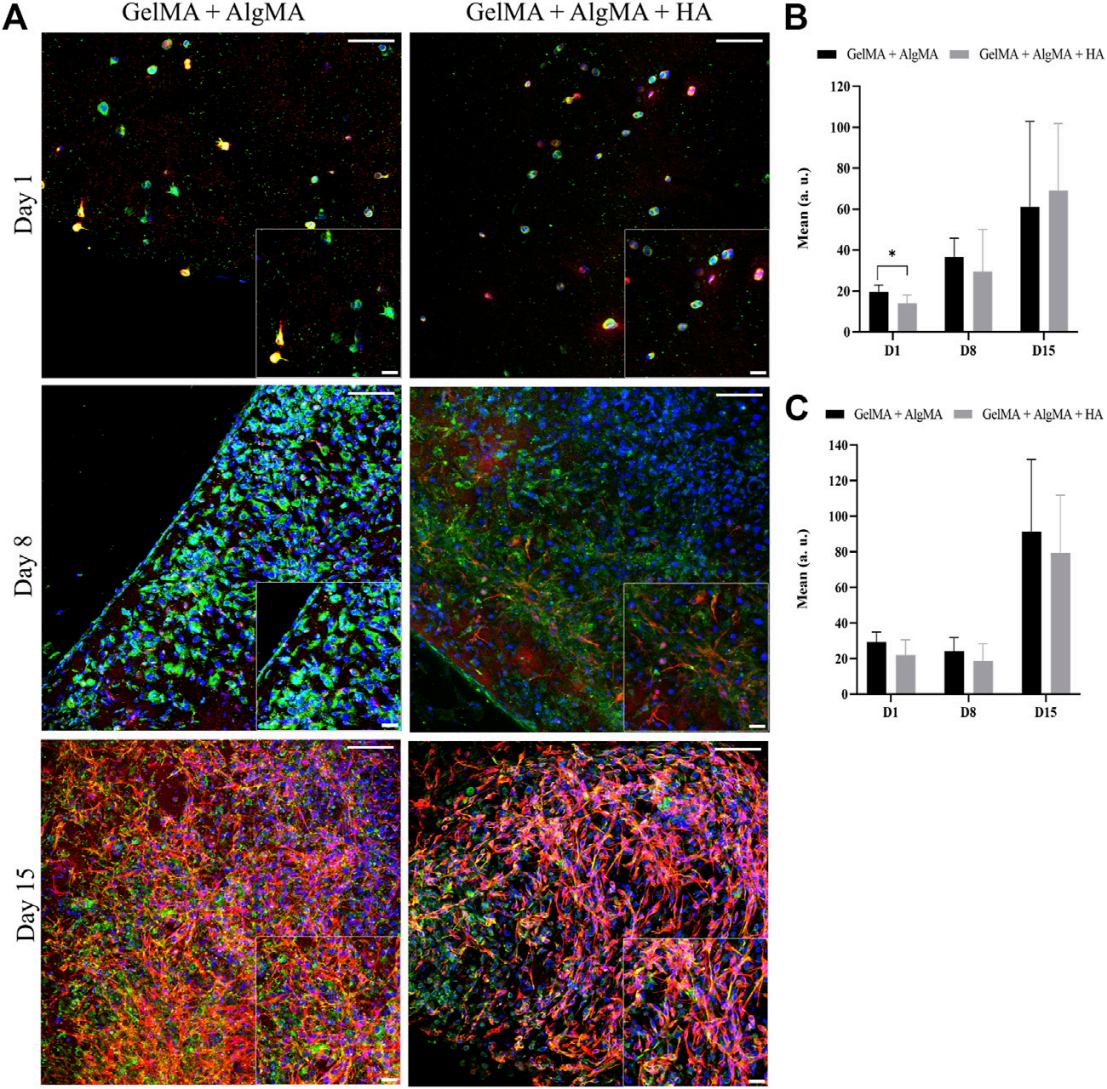
Evaluation of the differentiation of C17.2 cells embedded in the hydrogel after printing for up to 15 days. **(A)** Representative confocal images of immunofluorescence assay progenitor marker nestin is stained in green, neuron marker *β*-III tubulin is stained in red, and nuclei are stained in blue (scale bar 100 µm). Local zoom images are placed at the bottom right corner of each image with a scale bar of 30 µm. Quantification of the mean expression of nestin **(B)** and *β*-III tubulin **(C)**. Data are presented as mean ± standard deviation. Statistical significance was calculated using a two-way variance analysis (ANOVA) with Sídák’s *post hoc* multiple comparisons test considering *p < 0.05.

An immunofluorescence assay was then performed to assess the differentiation of the cells after printing at three time points (days 1, 8, and 15) (Figure 8A). At the first time point, 1 day after printing, most cells expressed nestin, and some also started expressing *β*-III tubulin. A low density of cells was again observed. The cells were dispersed through the biomaterials and presented a round shape. On day 8, an increase in the number of cells was observed in both formulations, which translated into an increase in the nestin expression, whereas the expression of *β*-III tubulin remained the same. The morphology of the cells was altered; the cells were more spread with small projections connecting the cells. On day 15, a slight increase in the number of cells was observed in the nucleus signal, accompanied by an increase in the nestin expression along with a substantial increase in the *β*-III tubulin expression (Figures 8B, C).

### Three-dimensional bioprinted construct allowed the maturation of culture with functional activity

**FIGURE 9:**
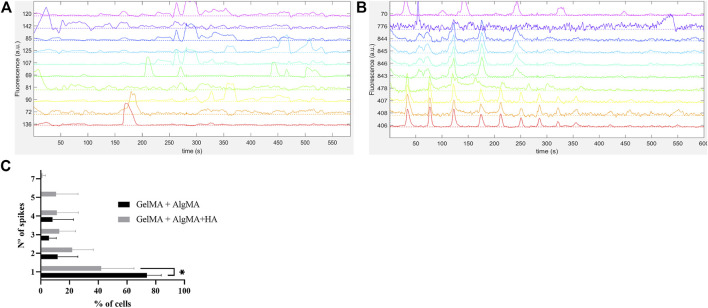
Functional assessment of the cells embedded in the biomaterials *via* calcium imaging with Fluo4AM **(A)** Traces of spontaneous activity of the cells embedded in GelMA + AlgMA hydrogel on day 15 of culture; **(B)** traces of spontaneous activity of the cells embedded in GelMA + AlgMA + HA hydrogel on day 15 of culture; **(C)** quantification of the number of spontaneous spikes of active cells in both hydrogels during 10 min of recording. Data are presented as mean ± standard deviation. Statistical significance was calculated using a two-way variance analysis (ANOVA) with Sídák’s *post hoc* multiple comparisons test considering *p < 0.05.

In addition to the expression of *β*-III tubulin, the functional activity of the cells is another indicator of neuronal maturation. As most of the expression of *β*-III tubulin was present on the last day of culture, we selected this time point to evaluate whether these cells could also demonstrate functional properties. Neuronal firing is indeed a sign of cell maturation as it requires the expression of specific cell-surface receptors that typically appear after the establishment of synaptic contacts. To assess the neuronal capability of eliciting action potentials, calcium imaging was performed, and traces of the recorded spontaneous activity are presented in Figure 9. Both formulations demonstrated spontaneous cell activity, with the formulation containing HA showing a higher frequency of spontaneous spikes during recording (Figures 9A, 9B). After classifying the percentage of cells with the number of spontaneous spikes per cell (from 1 to 7), it was evident that the GelMA + AlgMA + HA formulation had a percentage of cells with five and seven spikes per active cell that were not observed in the formulation without HA (Figure 9C). A significant difference (*p*-value = 0,0262) was observed in the percentage of cells with only one spike between formulations, with the control formulation presenting a higher value (73.9% ± 9.8% vs. 42.0% ± 22.8%). A difference in the distribution of the number of spikes was also noticeable when comparing the percentage of cells that had one, two, or more than two spikes (*p*-value = 0.0037), confirming an increase in the number of spontaneous spikes with GelMA + AlgMA + HA (Supplementary Figure S3).

## Discussion

In this study, the compatibility of a composite hydrogel with the culture and differentiation of NPCs was assessed, and this formulation was applied as a bioink for extrusion bioprinting. The development of 3D neuronal cultures still remains challenging because of the high sensitivity of neurons to changes in their surroundings (Brunetti et al., 2010). For our formulation, GelMA was selected owing its biocompatibility with neuronal culture and malleable mechanical and physical properties in combination with AlgMA. The last one was also biocompatible but has showed increased durability compared to GelMA because it was not degraded by the cells (Yue et al., 2015; Rastogi and Kandasubramanian, 2019). HA was also added to the formulation because it is one of the main components of the ECM in the brain (Sherman et al., 2002; Her et al., 2013). GelMA + AlgMA has been used with other cell types and has demonstrated high viability rates (Wei et al., 2015; García-Lizarribar et al., 2018; Tavafoghi et al., 2020). Hence, this formulation was used as the control.

The diffusion of water, nutrients, and gases is essential for the survival of cells in 3D scaffolds and their mobility within biomaterials. The swelling rate and porosity of the biomaterial are related, and when the swelling rates increase, an increase in the porosity is observed (Annabi et al., 2010). The control formulation (GelMA + AlgMA) presented values of the swelling rate, as previously reported by the group (García-Lizarribar et al., 2018), while our new formulation with HA had slightly higher values. This result is consistent with those in previous studies where HA formulations have been shown to increase the water uptake capability of scaffolds (Her et al., 2013). Stiffness is an important parameter of 3D biomaterials because it can directly affect the differentiation and activity of cells (Her et al., 2013; Wen et al., 2018). Materials with low compression values to simulate brain stiffness (approximately 1 kPa) lose definition quickly and degrade faster, which may be incompatible with long-term culture and 3D printing (Mahumane et al., 2018; Wang et al., 2019). Values within 1–10 kPa are considered optimal for neuronal models (Cadena et al., 2021). The results obtained in this study were within the range, with the GelMA + AlgMA + HA formulation reaching a slightly lower value than the control. This can be explained by the high water intake of HA, as previously mentioned. In addition, the degradation rate is an important feature to be evaluated because degradation can help neuronal cells to spread and move across the material or even control the differentiation of neuronal progenitor cells (Kharkar et al., 2013; Madl et al., 2017). The results showed that both materials were degraded over time. The biomaterial with HA showed a slightly longer degradation time, which could be beneficial for long-term assays, allowing more time for cells to adapt to the environment and start producing their own ECM.

Based on the SEM results, the porosity of the surface of our formulation and the control was visible; both showed a high frequency of pores. A smaller pore size was observed in the formulation with HA, which could be attributed to the complexity of the formulation. In other studies, the SEM images of biomaterials with similar compositions have presented much larger pores (Her et al., 2013; Noshadi et al., 2017; Krishnamoorthy et al., 2019). These differences could be attributed to the processing of the samples for SEM analysis. Most studies performed freeze-drying before SEM analysis, and this process can induce the production of larger pores due to the formation of ice crystals (Annabi et al., 2010; Santana et al., 2015; Noshadi et al., 2017). A high swelling rate, in combination with smaller pore size, could also be an indicator of water absorption by the pore walls (Zhao et al., 2010). Another important point is that with the degradation of the biomaterial during culture, the size of the pore is set to increase. With an increase in the pore size, cells tend to demonstrate higher extrusion of filopodia (Kim et al., 2018). This finding could be observed in the immunostaining images of these formulations, whereby on day 15, the cells presented larger projections and connectivity (Figure 5A) as well as in the SEM images (Figure 3), where the cell number and connectivity increased on day 15.

Biological assays were performed before printing to evaluate the compatibility of the bioink with neuronal cell cultures and differentiation. The formulation with HA was compatible with long-term culture (28 days), with viability rates similar to those of the control formulation. Cell proliferation was visible in the increased number of cells from days 1 to 15 of culture. Mammalian cells usually proliferate until they reach a state of proximity during which they stop division, cell growth, and motility (Gu et al., 2017). NPCs, including C17.2 cells, also proliferate until they enter the post-mitotic phase during differentiation (Bechara et al., 2011; Wang et al., 2014). Differentiation was also possible in the composite formulation, with the stable nestin expression and increased *β*-III tubulin expression. These results indicate that the C17.2-derived NPC populations mature at different rates. One subpopulation maintains an immature state, whereas the other subpopulation of NPCs undergoes neural differentiation and maturation. However, the decrease in PAX6 and DLX on day 28, specifically for NPCs cultured on GelMA + AlgMA + HA, is consistent with a large differentiation of NPCs. In addition, significant differences in the expression of the neuronal marker *β*-III tubulin were observed at day 21, where GelMA + AlgMA + HA presented a significant increase in the expression of *β*-III tubulin compared to the control (Figures 5B, C), validating the suitability of our formulation for neuronal culture and differentiation. The gene expression observed up to day 15 was consistent with the results of immunofluorescence assays for the expression of the two aforementioned markers. The results were complemented by evaluating the expression of MAP2 and S100b, which increased by day 15. These two markers indicated that part of the population was indeed differentiated into mature neurons, but astrocytes were also present. C17.2 cells are described to differentiate into a mixed population of neurons and astrocytes (Lundqvist et al., 2013; Attoff et al., 2016). However, in the formulation with HA, the expression of S100b was lower than that in the formulation without HA, suggesting a smaller population of astrocytes than neurons, which could probably be related to the better spontaneous activity observed. However, by day 28, the expression of most differentiation-related genes was reduced compared to that on day 15, which could be an indication of degeneration of differentiated C17.2 cells by that late time point. This resembled the decrease in *β*-III tubulin and MAP2 levels reported in a previous study (Wang et al., 2014). Nevertheless, analyzing the *β*-III tubulin values at day 28 showed that the GelMA + AlgMA + HA composition demonstrated a slowed degeneration process compared with hydrogels without HA. The degeneration of differentiated neuronal cells may be attributed to an unfavorable environment for the maintenance of neuronal cells or a lack of sufficient cell networks that are necessary for long-term neuronal survival *in vitro*. The degradation of the hydrogels in both formulations was low until day 28. The interaction between neuronal cells seemed to be enhanced at day 28 with neuronal cluster formation (Figures 4A, 4B), and the environmental conditions were not significantly modified, suggesting that modification of the media conditions between days 15 and 28 may be necessary to maintain the differentiation process.

Extrusion bioprinting is one of the most commonly used bioprinting methods; however, cell viability can be affected by shear stress during printing (Nair et al., 2009). After optimization of the printing parameters, it was possible to print C17.2 cells in a defined 10-mm square grid (Figure 6A). The viability rates were higher than 80% after printing and in the following 2 weeks (Figure 6C), indicating that the formulations could be printed with a good definition without damaging the cells. Throughout the culture, cells proliferated until the last time point when they appeared to reach confluency, identical to the increased proliferation observed in previous studies (Figure 4C). A change in the morphology of the cells was distinguishable. The cells were in round shape on the day after printing and small projections after 1 week. Subsequently, an increase in those projections and cell connectivity was observed at the last time point (Figure 7A). A change in the size of the nuclei was also noticed, from being characteristically large in the case of NPCs to decreasing over time, indicating the onset of the process of differentiation (Lee et al., 2010). The expression of nestin was constant throughout the 2 weeks, whereas the expression of *β*-III tubulin increased over time, especially by day 15, in agreement with previous results.

In addition to compatibility, the matrix of a neuro environment needs to be electrically conductible to allow the formation of a mature neuronal network (Cadena et al., 2021). A calcium imaging assay was performed at the last time point to assess the presence of cell activity within the bioprinted materials. Despite the challenges in recording the activity of neuronal networks in 3D, spontaneous activity was detected on both hydrogels. GelMA + AlgMA + HA outperformed the control formulation, with a higher number of spontaneous spikes of activity. The increase in the number of spikes corresponding to spontaneous activity is a characteristic of the maturation of neuronal culture (Chiappalone et al., 2006). This suggests that our formulation was capable of supporting the culture of a mature neuronal network.

In combination with the previous results, GelMA + AlgMA + HA was proven as a suitable hydrogel for extrusion bioprinting, allowing the printing of a defined structure with high cell viability and supporting differentiation into mature neurons.

## Conclusion

Finding a suitable material for printing and culturing neural cells remains a challenge. In this study, the compatibility of the GelMA + AlgMA + HA hydrogel as a bioink to print NPCs and sustain their subsequent differentiation was assessed. Based on the optimization results, GelMA + AlgMA + HA was compatible with the culture with high viability rates, and differentiation of NPCs with increased expression of *β*-III tubulin and MAP2 was observed up to day 15 of culture. GelMA + AlgMA + HA also demonstrated a high water intake, slower degradation, and compression modulus, resulting in defined structures when used as a bioink for extrusion bioprinting. The cells within the bioprinted construct presented a higher viability rate while maintaining their capacity for differentiation into functional neurons, rendering this formulation a promising biomaterial for the proliferation and differentiation of mNPC. The long-term maintenance of the differentiated culture requires further studies considering that the formulation with HA seems to slow the degeneration process observed.

## Data Availability

The original contributions presented in the study are included in the article/Supplementary Material; further inquiries can be directed to the corresponding authors.

## Supplementary material

Supplementary Figure S1 Optimization of the polymer concentration through viability and immunofluorescence assays. **(A)** Representative confocal images of the viability assays of different polymer concentrations with 3% GelMA, 0–0.5% AlgMA, and 2.5–5% HA. Nuclei are stained blue, calcein AM in green represents the live cells, and EthD-1 in red represents the dead cells (scale bar 100 µm). **(B)** Representative confocal images of immunofluorescence assay of different polymer concentrations with 3% GelMA, 0–0.5% AlgMA, and 2.5–5% HA. Progenitor marker nestin is stained in green, neuron marker β-III tubulin is stained in red, and nuclei are stained in blue (scale bar 100 µm). **(C)** Biomaterial drops on days 1 and 7 of the culture. Black arrows point to the less visible drops (scale 2.5 mm).

Supplementary Figure S2 Optimization of the printing parameters. **(A)** Images of the printing scaffolds for the three conditions and both hydrogel formulations (scale bar 2.5 mm). **(B)** Measurement of the filament width of the structures printed under the three optimization conditions. Data are presented as mean ± standard deviation. Statistical significance was calculated using two-way variance analysis (ANOVA) with Turkey’s and Sídák’s *post hoc* multiple comparisons test considering **p<0.01; ***p<0.001; ****p<0.0001.

Supplementary Figure S3 Representation of the quantification of the number of spikes separated by the percentage of cells that presented one, two, or more than two spikes. Data are presented as mean ± standard deviation.

Supplementary Table S1 Conditions of optimization of the printing process.
