# Supplementary material for: Hyaluronic acid-based bioink improves the differentiation and network formation of neural progenitor cells
Source: Front Bioeng Biotechnol. 2023 Mar 3;11:1110547. doi: 10.3389/fbioe.2023.1110547 (PMC10020230; doi:10.3389/fbioe.2023.1110547)
Supplement: Supplementary file 1 [file DataSheet1.docx]

**Supplementary Information**


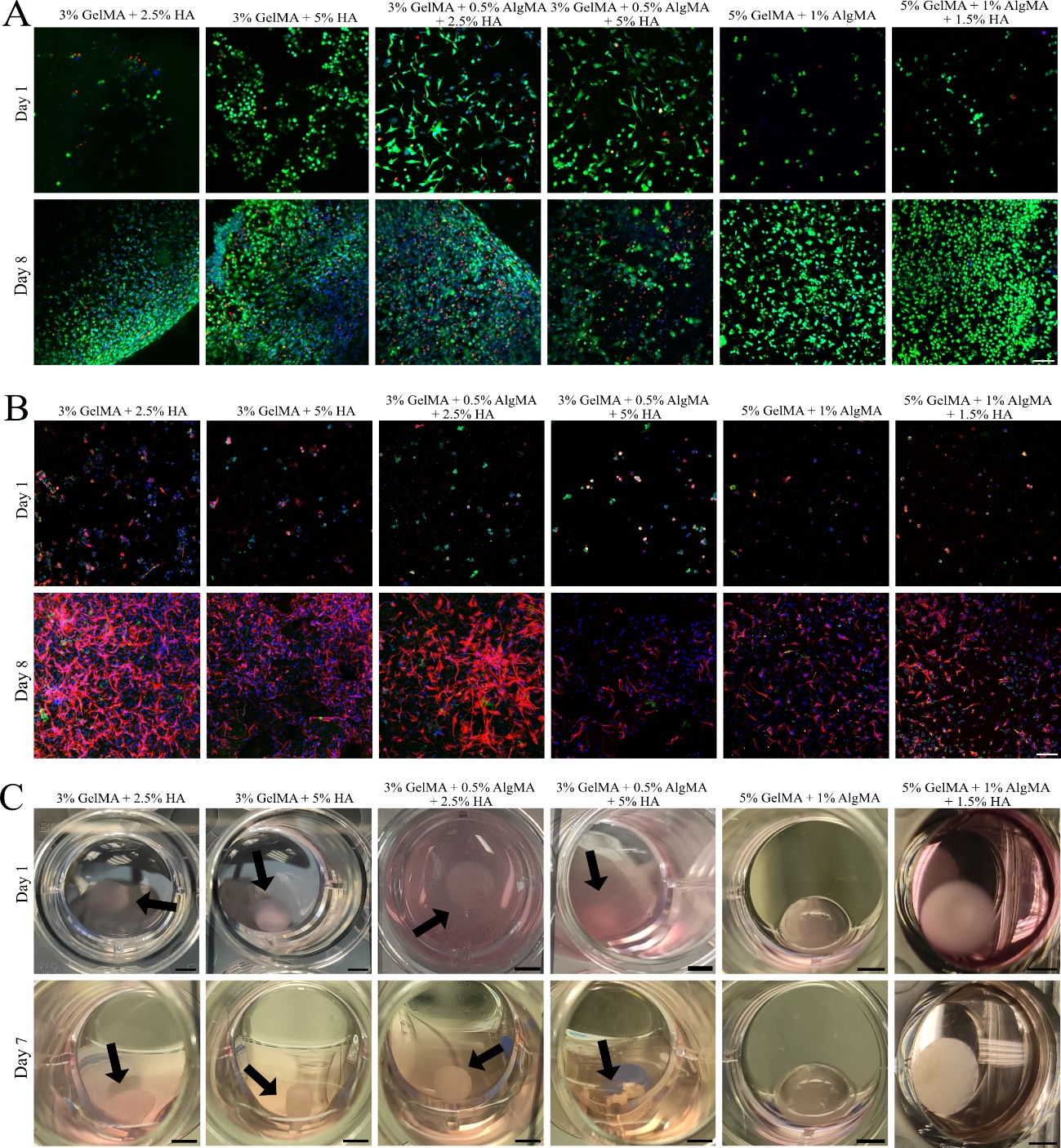


Figure S1 Optimization of the polymer concentration through viability and immunofluorescence assays. (A) Representative confocal images of the viability assays of different polymer concentrations with 3% GelMA, 0–0.5% AlgMA, and 2.5–5% HA. Nuclei are stained blue, calcein AM in green represents the live cells, and EthD-1 in red represents the dead cells (scale bar 100 μm). (B) Representative confocal images of immunofluorescence assay of different polymer concentrations with 3% GelMA, 0–0.5% AlgMA, and 2.5–5% HA. Progenitor marker nestin is stained in green, neuron marker β-III tubulin is stained in red, and nuclei are stained in blue (scale bar 100 μm). (C) Biomaterial drops on days 1 and 7 of the culture. Black arrows point to the less visible drops (scale 2.5 mm).


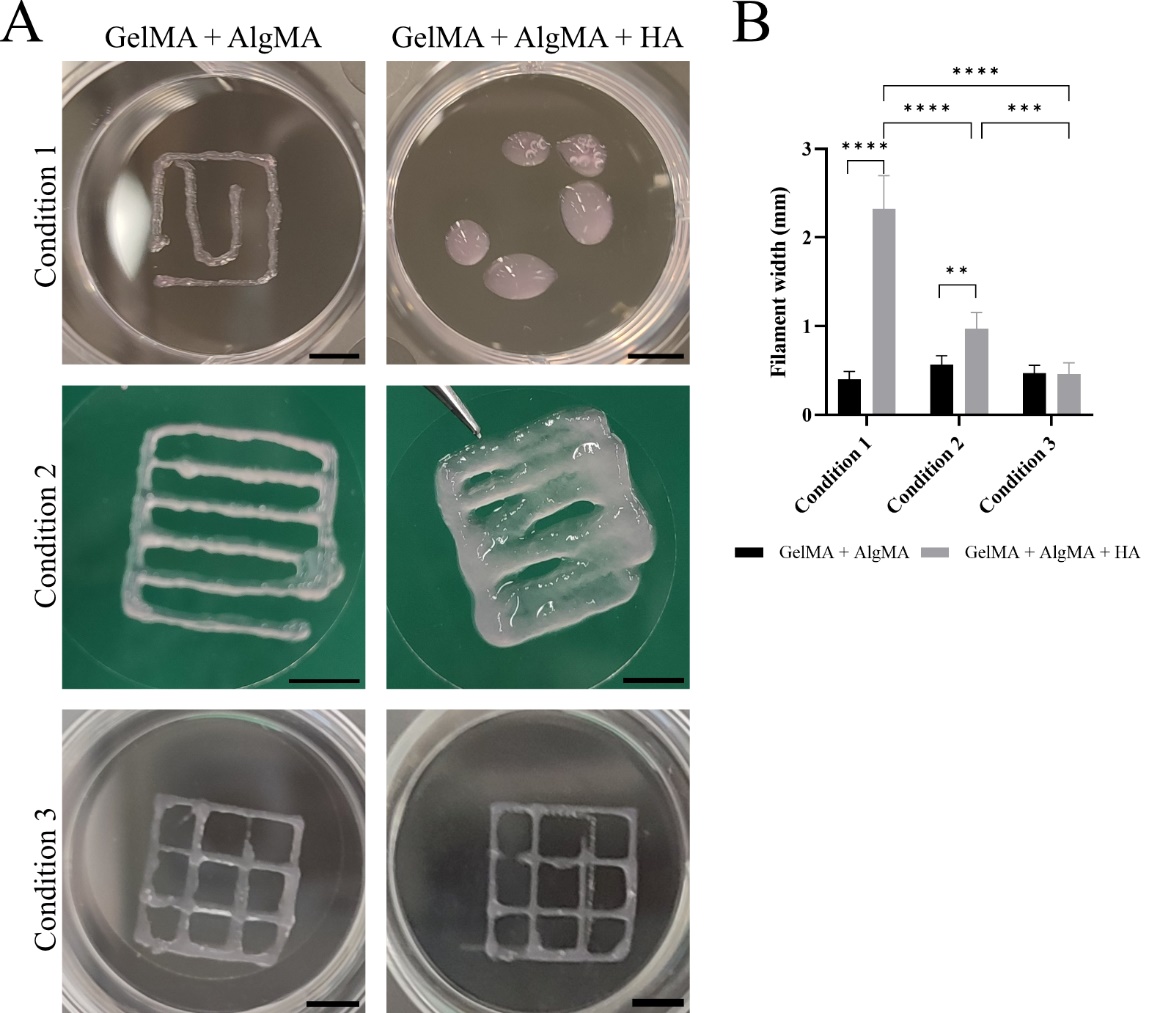


Figure S2 Optimization of the printing parameters. (A) Images of the printing scaffolds for the three conditions and both hydrogel formulations (scale bar 2.5 mm). (B) Measurement of the filament width of the structures printed under the three optimization conditions. Data are presented as mean ± standard deviation. Statistical significance was calculated using two-way variance analysis (ANOVA) with Turkey’s and Sídák’s post hoc multiple comparisons test considering **p<0.01; ***p<0.001; ****p<0.0001.

Table 1 Conditions of optimization of the printing process.

|  | Pressure (Pa) | Printing speed (mm/s) | Temperature (°C) |
| --- | --- | --- | --- |
| Condition 1 | 2 | 3 | 12 |
| Condition 2 | 2 | 3 | 18 |
| Condition 3 | 2 | 30 | 18 |


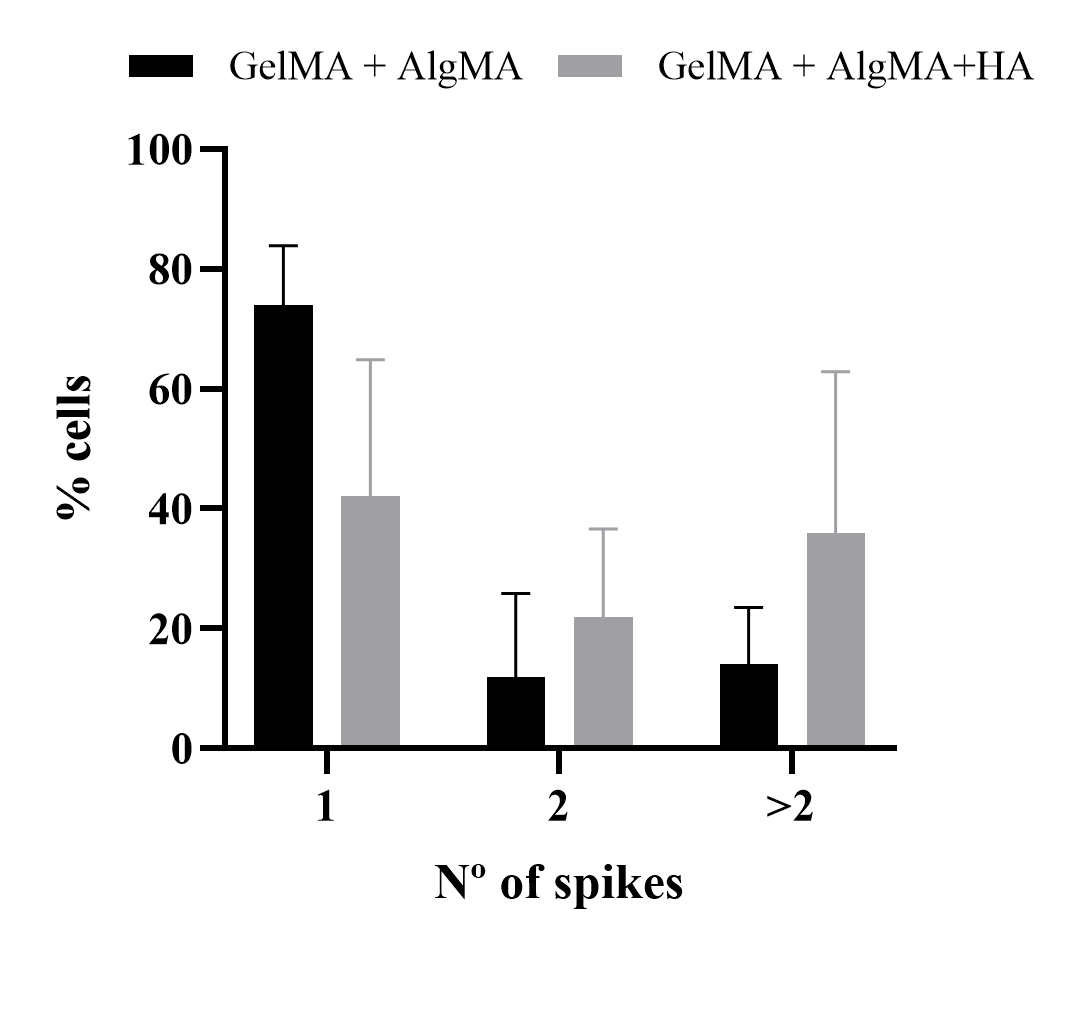


Figure 3 Representation of the quantification of the number of spikes separated by the percentage of cells that presented one, two, or more than two spikes. Data are presented as mean ± standard deviation.
